# Nodular Scleroderma in a Patient With Anti–RNA Polymerase III–Antibody Positive Systemic Sclerosis: A Case Report and Literature Review

**DOI:** 10.1111/1346-8138.70391

**Published:** 2026-07-12

**Authors:** Kyoko Kanno, Yuki Kobayashi, Yasuyuki Fujita

**Affiliations:** ^1^ Department of Dermatology Asahikawa Medical University Asahikawa Japan

**Keywords:** anti–RNA polymerase III antibody, keloidal lesion, nodular scleroderma, PUVA therapy, systemic sclerosis

## Abstract

Nodular scleroderma (NS) is a rare manifestation of systemic sclerosis (SSc) that can clinically resemble keloids, often leading to diagnostic difficulty. Herein we report a case of a 76‐year‐old woman with anti–RNA polymerase III antibody–positive SSc who developed progressive erythematous, pruritic, keloid‐like plaques 3 months after rituximab therapy. Histopathology showed thickened collagen bundles extending from the superficial to mid‐dermis, increased fibroblasts, mucin deposition, and reduced elastic fibers without the hyalinized collagen characteristic of keloids, confirming NS. To date, NS associated with anti‐RNA polymerase III antibody‐positive SSc has not been reported. The patient was treated with topical PUVA, resulting in reduction of pruritus and softening of nodular lesions. Review of Japanese and international cases indicates that NS commonly arises during active SSc, often in areas of mechanical stress, and shows variable therapeutic response. This case expands the serological spectrum of NS and supports PUVA as a therapeutic option for symptomatic lesions.

## 1 Introduction

Nodular scleroderma (NS) is a rare dermatologic manifestation characterized by keloid‐like or nodular lesions that occur either as a variant of localized scleroderma or in association with systemic sclerosis (SSc). Fewer than 60 cases have been reported in the literature. NS arising in the context of SSc typically appears during the active phase of the disease and may be triggered by mechanical stress or injury. Here, we describe a patient with anti‐RNA polymeraseIII antibody positive SSc who developed NS, and the NS lesions responded partially to topical psoralen plus ultraviolet A (PUVA) phototherapy.

## 2 Case Report

A 76‐year‐old woman previously diagnosed with anti–RNA polymerase III antibody–positive systemic sclerosis in 2021 presented with progressive skin induration. Her medical history included scleroderma, renal crisis, and gastroesophageal reflux disease, but not keloid formation.

Pruritic erythema had already appeared 8 months prior to the initiation of rituximab therapy, and although topical corticosteroids were administered, the response was poor.

Considering the patient's overall condition, the patient received rituximab at a reduced dose of 350 mg/m^2^ weekly for 4 weeks to treat diffuse cutaneous involvement of SSc.

Three months later, erythematous pruritic plaques appeared on the anterior chest. The lesions gradually became linear and raised, clinically resembling keloids. Despite receiving three cycles of rituximab and topical corticosteroids, the rash did not improve.

Multiple linear sclerotic plaques accompanied by fresh‐red erythema and severe itch were distributed in sequence on the anterior chest and upper back (Figure 1a). Similar rashes were also observed on the buttocks, inner thighs, and knee joints. The distribution of the keloid‐like rashes is shown in the diagram (Figure 1b).

**FIGURE 1:**
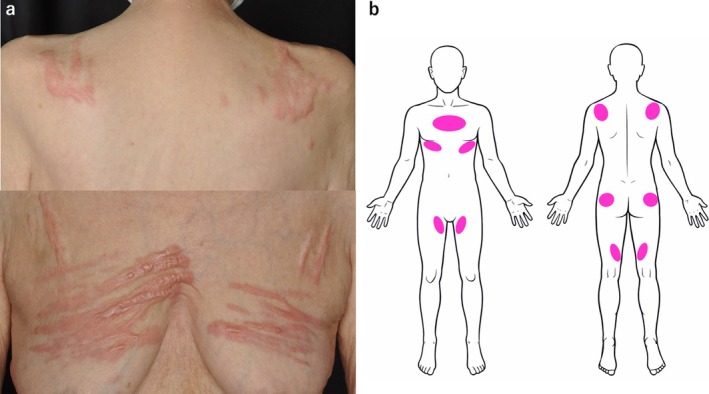
Initial clinical manifestations. (a) Keloid‐like lesions on the chest and upper back. (b) Distribution map of the lesions.

Given the background of the diffuse cutaneous SSc, the Modified Rodnan Skin Score (mRSS) was 34, indicating very severe skin involvement. Raynaud phenomenon, periungual capillary changes, sclerodactyly, and limited range of motion in the hands, feet, and trunk were noted. Laboratory findings reveal that antinuclear antibody was positive at ×80 (speckled/homogeneous), and that anti–RNA polymerase III antibody was markedly elevated (> 790 U/mL). Other SSc related antibodies, including anti‐Scl‐70 antibody and anti‐centromere antibody, were negative. Routine blood tests were within normal limits except for mild anemia.

Histopathological examination of the plaque revealed thickened collagen bundles, with a relatively clear border extending from the superficial to the mid‐dermis. Adnexal structures, including sweat glands, were preserved in the lesional dermis. Mild inflammatory cell infiltration was observed around superficial dermal vessels (Figure 2a). Collagen fibers were markedly increased, with diffuse proliferation of dermal fibroblasts. The thick, hyalinized collagen fibers characteristic of keloids were not observed (Figure 2b). CD34 immunostaining was performed and demonstrated a reduction of CD34‐positive fibroblasts within the nodule (Figure 2c). On Elastica van Gieson staining, a decrease and rupture of elastic fibers in the lesion were seen (Figure 2d). Colloid iron staining confirmed the deposition of mucin between the collagen fibers (Figure 2e).

**FIGURE 2:**
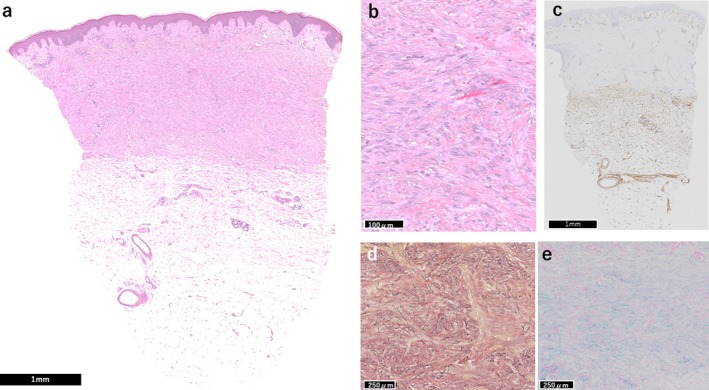
Histopathology of the elevated lesion on the chest. (a) Thickened and demrcated collagen bundles extending from the superficial to the mid‐dermis. (b) Higher magnification revealing the absence of thick, hyalinized collagen fibers usually observed in keloids. (c) CD34 staining demonstrating a reduction of CD34‐positive fibroblasts within the nodule. (d) Colloidal iron staining revealing mucin deposition. (e) Elastica van Gieson staining demonstrating reduction and rupture of elastic fibers.

Based on these clinical and histological findings, we diagnosed her with linearly distributed NS associated with systemic sclerosis.

There was no improvement after treatment with superpotent topical steroids and 8 sessions of narrowband ultraviolet B therapy. Thereafter, topical PUVA therapy was administered as 10 daily sessions per cycle, with two cycles separated by 1 month. The irradiation dose started at 300 mJ/cm^2^ and gradually increased up to 500 mJ/cm^2^. Pruritus score evaluated using the numerical rating scale decreased from 10/10 pre‐treatment to 8/10 after the first cycle and to 7/10 after the second cycle. Nodular lesions softened and became less erythematous over time (Figure 3a,b).

**FIGURE 3:**
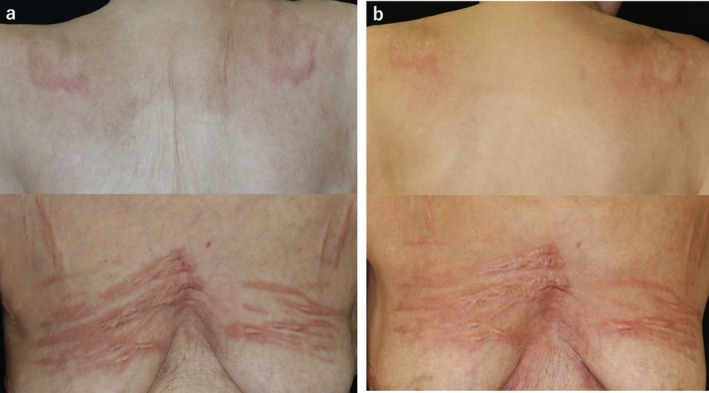
Clinical appearance of the keloid‐like lesion after UVA phototherapy combined with topical methoxsalen ointment (0.3%). Two 10‐day treatment cycles were administered, separated by a 1‐month interval. (a) After the first treatment cycle. (b) After the second treatment cycles. Erythema and elevation mildly improved, especially on the shoulders.

## 3 Discussion

NS is a rare manifestation of morphea in the background of localized or systemic sclerosis which remains poorly understood. Its clinical characteristics resemble keloids or hypertrophic scars; however, most patients have no preceding trauma, and lesions often appear during periods of active SSc. Histopathological findings generally include thickened collagen fibers, with an irregular arrangement, fibroblast proliferation, and mucin deposition, without the thick hyalinized collagen typical of keloids. These intermediate histopathological features help distinguish NS from other fibrosing disorders [1].

To the best of our knowledge, 58 cases of NS have been reported worldwide, including twelve cases from Japan. Patients ranged from 13 to 76 years of age, with a strong female predominance. 50 of the 58 NS cases (86.2%) occurred in patients with SSc, most commonly within 6 months of SSc onset or disease flare [2, 3, 4]. This is notably given that contrasts with only 2.4%–7.4% of patients with SSc developed localized scleroderma [5]. Lesions were described as nodules, thickened red plaques with irregular margines, and hand‐sized tuberosities. Although a linear presentation of NS has been reported [6], cases with multiple linear sclerotic lesions such as those seen in patient, have not been described.

Raynaud's phenomenon was observed in 47 of 53 evaluable NS cases with SSc, consistent with the present case (88.7%). Raynaud phenomenon status was not reported in 3 cases. ANA positivity is common in NS, but staining patterns vary, with speckled and homogeneous patterns being the most frequently described. While anticentromere antibody is most frequently detected in localized scleroderma (5 cases 8.9%), anti‐Scl‐70 antibody is most frequently positive in NS (11cases, 19.6%). NS cases tend to lack autoantibodies specific to disease onset; to date, this is the first reported NS case with anti‐RNA polymeraseIII antibody (Table in Supplement) (2–4).

Even though the pathogenesis of NS remains unclear, several mechanisms have been proposed, including fibrosis‐prone microenvironments during the active phase of SSc and mild mechanical stimuli such as scratching. In our case, the presence of pruritus and the act of scratching were considered background contributing factors. Furthermore, the predilection for pressure‐prone areas supports the possibility that the lesions developed along sites of scratching [7, 8]. In addition, anti‐RNA polymerase III antibody–positive SSc is often severe and prone to repeated episodes of high disease activity, which may lead to hyperreactivity to mechanical scratching, resulting in the characteristic distribution of NS in our case.

An additional noteworthy aspect of this case is that the nodular lesions developed during rituximab therapy. To the best of our knowledge, similar cases have not been previously reported. Although a causal relationship cannot be established, this temporal association raises the possibility of a mechanistic link. Rituximab‐induced B‐cell depletion may lead to paradoxical immune modulation, including alterations in cytokine balance and loss of regulatory B‐cell function, which could potentially enhance profibrotic pathways. Furthermore, B cells are known to interact with fibroblasts through cytokines such as IL‐6 and TGF‐β; thus, their depletion may indirectly dysregulate fibroblast activity and promote localized collagen overproduction [9]. In the context of highly active systemic sclerosis and repeated mechanical stimuli such as scratching, these immunological changes might contribute to aberrant wound healing responses, resulting in the development of nodular scleroderma‐like lesions.

The differential diagnosis of NS includes keloids and hypertrophic scars. Although nodular scleroderma closely resembles keloids clinically, the absence of prominent keloidal collagen, and the presence of reduced, fragmented elastic fibers and diffuse mucin deposition, all uncommon in keloids. Favored a diagnosis of nodular scleroderma suggested a fibrosing process related to SSc rather than true keloid formation in our case.

Treatment remains challenging, as no standardized therapeutic approach has been established. Therapies such as topical or intralesional corticosteroids, tacrolimus, vitamin D analogs, systemic immunosuppressants, and various phototherapies have been used with variable success. Phototherapy, including PUVA and UVA1, appears to be among the more effective modalities because of its capacity to reduce collagen synthesis and modulate profibrotic cytokines [4, 10, 11]. In the present case, topical PUVA provided symptomatic relief and softening of lesions, aligning with previously reported therapeutic responses.

Overall, NS represents a distinct, albeit rare, fibrosing phenotype within scleroderma. NS may emerge following changes in disease activity. Early intervention, with careful histological distinction from keloids, may improve patients' quality of life.

## Ethics Statement

The authors have nothing to report.

## Consent

Written informed consent for publication of the clinical details and images was obtained from the patient.

## Conflicts of Interest

The authors declare no conflicts of interest.

## Supporting information


**Table S1:** Comparison between localized scleroderma associated with SSc and nodular scleroderma (NS) with SSc.

## Data Availability

The data that support the findings of this study are available on request from the corresponding author. The data are not publicly available due to privacy or ethical restrictions.
