# Supplementary material for: Nodular Scleroderma in a Patient With Anti–RNA Polymerase III–Antibody Positive Systemic Sclerosis: A Case Report and Literature Review
Source: J Dermatol. 2026 Jul 12;53(8):1213–6. doi: 10.1111/1346-8138.70391 (PMC13434983; doi:10.1111/1346-8138.70391)
Supplement: Supplementary file 1 [file JDE-53-1213-s001.docx]

**Supplementary Table S1. Comparison between localized scleroderma associated with SSc and nodular scleroderma (NS) with SSc**

| **Clinical features** | **Localized scleroderma + SSc** | **NS + SSc (including our case)** |
| --- | --- | --- |
| Frequency | 2.4–6.7% of SSc patients | 56 cases |
| ANA positivity | 67% | 42 (82%) |
| Anti-centromere antibody | 28% | 5 (8.9%) |
| Anti-Scl-70 antibody | 18% | 11(19.6%) |
| Anti-RNAP III antibody | 8% | 1 (0.18%)* |
| Raynaud’s phenomenon | Almost all cases | 47 (88.7%) |
|  |  |  |

* Observed only in our case.
